# Dysregulated Nephrin in Diabetic Nephropathy of Type 2 Diabetes: A Cross Sectional Study

**DOI:** 10.1371/journal.pone.0036041

**Published:** 2012-05-17

**Authors:** Belinda Jim, Mythili Ghanta, Andi Qipo, Ying Fan, Peter Y. Chuang, Hillel W. Cohen, Maria Abadi, David B. Thomas, John Cijiang He

**Affiliations:** 1 Division of Nephrology, Department of Medicine, Jacobi Medical Center, Albert Einstein College of Medicine, Bronx, New York, United States of America; 2 Department of Nephrology, Shanghai Jiao Tong University Affiliated Sixth People's Hospital, Shanghai, China; 3 Division of Nephrology, Department of Medicine, Mount Sinai School of Medicine, New York, New York, United States of America; 4 Department of Epidemiology and Population Health, Albert Einstein College of Medicine, Bronx, New York, United States of America; 5 Department of Pathology, Jacobi Medical Center, Albert Einstein College of Medicine, Bronx, New York, United States of America; 6 Nephrocor, Uniondale, New York, United States of America; 7 Division of Nephrology, Department of Medicine Bronx, James J. Peters VA Medical Center, New York, United States of America; University of Florida, United States of America

## Abstract

**Background:**

Podocyte specific proteins are dysregulated in diabetic nephropathy, though the extent of their expression loss is not identical and may be subject to different regulatory factors. Quantifying the degree of loss may help identify the most useful protein to use as an early biomarker of diabetic nephropathy.

**Methodology/Principal Findings:**

Protein expression of synaptopodin, podocin and nephrin were quantified in 15 Type 2 diabetic renal biopsies and 12 control patients. We found statistically significant downregulation of synaptopodin (P<0.0001), podocin (P = 0.0002), and nephrin (P<0.0001) in kidney biopsies of diabetic nephropathy as compared with controls. Urinary nephrin levels (nephrinuria) were then measured in 66 patients with Type 2 diabetes and 10 healthy controls by an enzyme-linked immunosorbent assay (Exocell, Philadelphia, PA). When divided into groups according to normo-, micro-, and macroalbuminuria, nephrinuria was found to be present in 100% of diabetic patients with micro- and macroalbuminuria, as well as 54% of patients with normoalbuminuria. Nephrinuria also correlated significantly with albuminuria (*rho* = 0.89, p<0.001), systolic blood pressure (*rho* = 0.32, p = 0.007), and correlated negatively with serum albumin (*rho* = −0.48, p<0.0001) and eGFR (*rho* = −0.33, p = 0.005).

**Conclusions/Significance:**

These data suggest that key podocyte-specific protein expressions are significantly and differentially downregulated in diabetic nephropathy. The finding that nephrinuria is observed in a majority of these normoalbuminuric patients demonstrates that it may precede microalbuminuria. If further research confirms nephrinuria to be a biomarker of pre-clinical diabetic nephropathy, it would shed light on podocyte metabolism in disease, and raise the possibility of new and earlier therapeutic targets.

## Introduction

Diabetes affecting the kidney, or diabetic nephropathy (DN), affects approximately one third of patients with either Type 1 or Type 2 diabetes mellitus [1]. Given the epidemic of new patients projected to have diabetes by year 2050, the prevalence of DN will rise just as dramatically [1]. Thus, the only feasible way to tackle this health care crisis is by prevention of disease with early detection.

Small amounts of albumin in the urine, or microalbuminuria is the current early biomarker. However, its association with progression to renal failure is unclear, as microalbuminuria does not always lead to progressive renal failure [2]. Furthermore, it is found in other disease states such as urinary tract infection [3] and hemodynamic stress (exercise, fever, congestive heart failure) [4], [5].

We now know that much of the early inciting events stem from podocyte pathology. The podocyte is a specialized visceral epithelial cell that helps to establish the glomerular filtration barrier and prevents protein loss, along with the glomerular basement membrane and the endothelial cell layer. Occurrence of podocytopenia (decreased number) and podocyturia (podocytes in urine) in DN are well established [6]–[8]. Podocyte loss initiates the process of glomerulosclerosis by accelerating synechiae between podocytes and the glomerular basement membrane. Both the highly specialized cytoskeleton and its complex slit diaphragm contribute to the glomerular filtration barrier. Derangement of either aspect leads to proteinuria [9]–[11]. In DN, altered expression of podocyte specific proteins such as synaptopodin [12], podocin [13]–[15] and nephrin have been described [16]–[18].

Synaptopodin, a proline rich protein, directly interacts with the α-actinin-induced actin filaments. Downregulation of synaptopodin expression leads to structural and functional changes such as loss of stress fibers, aberrant formation of filopodia, and impaired cell migration [19], [20]. Nephrin and podocin, on the other hand, are slit-diaphragm associated proteins. Nephrin, being a transmembrane protein with an extracellular and intracellular domain, forms the scaffolding of the podocyte slit diaphragm. It is linked to the actin cytoskeleton via podocin and CD2AP. These proteins not only characterize the differentiated phenotype of the podocyte but have also been identified to have functional characteristics as they interact with the PI3K/AKT signaling pathway to maintain functional integrity [21]. Mutation of either protein can result in foot process effacement and massive proteinuria [22], [23]. Given their dysregulation in DN, podocytes and their specific proteins pose as attractive candidates as either diagnostic or predictor biomarkers of disease. Patari et al. has described presence of nephrin in the urines of Type I diabetic patients even in the absence of microalbuminuria [24], while Nakamura et al. discovered urinary podocytes only in patients with micro- and macroalbuminuria, not normoalbuminuria [8]. However, there is little information in Type 2 diabetic patients, which make up the majority of patients who progress to end-stage renal disease.

In the current study, we investigated the morphologic alterations of podocyte-specific proteins in DN biopsies from patients with Type 2 diabetes and found significant downregulation of synaptopodin, podocin and nephrin expression in the diabetic group as compared to controls. Given the availability of a reliable method of quantifying nephrin in the urine (nephrinuria), we measured its levels in diabetic patients and found that it was detected in 54% of normoalbuminuric patients, suggesting its potential role as an early biomarker.

## Methods

### Objectives

Our goal is to study the expression of podocyte specific proteins in renal biopsies of Type 2 diabetic patients, and then to select the most downregulated protein as a marker for early detection of renal disease.

### Participants

#### Biopsy samples

Study samples consisted of kidney biopsies of 15 DN patients that were obtained at Jacobi Medical Center in Bronx, New York, USA and archived at Columbia-Presbyterian Hospital in New York, New York, USA. Indications for biopsy included: presence of nephrotic syndrome without retinopathy, unexplained acute kidney injury, or presence of hematuria. All clinical data at time of biopsy were retrieved from electronic medical records at Jacobi Medical Center. Twelve control biopsies consisted of normal kidney tissue from tumor nephrectomies. This study has been approved by the Internal Review Board of the Albert Einstein College of Medicine.

#### Urine collection

Patients were selected from the outpatient nephrology clinic at Jacobi Medical Center. A one-time random urine sample was collected by the clean catch method on all study and control subjects. Inclusion criteria for study patients were: history of type 2 diabetes, absence of another renal or urinary tract disease, and absence of pregnancy. There were no exclusion criteria based on race, age, or gender. Diabetic patients were categorized as having normoalbuminuria when urine albumin-to-creatinine ratio (UACR) <30 mg/g, microalbuminuria when UACR was between 30 and 300 mg/g, and macroalbuminuria when UACR was >300 mg/g. All control subjects were healthy volunteers from the medical staff with no history of diabetes, hypertension, or renal disease. Demographic and clinical data were recorded, including age, sex, weight, height, medications, blood pressure, blood urea nitrogen (BUN), serum creatinine, Hb_A1C_, random urine albumin, urine creatinine, and urine protein. All clinical laboratories values were measured at the Department of Clinical Laboratories at Jacobi Medical Center. Estimated glomerular filtration rate (eGFR) was calculated by the 4-variable Modification of Diet in Renal Disease study equation [25]. This study has been approved by the Internal Review Board of the Albert Einstein College of Medicine.

### Description of Procedures or Investigations undertaken

#### Immunohistochemistry

Paraffin-embedded tissues sections were deparaffinized in microwave oven for 3 minutes. The sections were rehydrated in graded series of xylene and alcohols. Endogenous peroxidase activity was blocked by 3% hydrogen peroxide in distilled water for 15 minutes. Antigen retrieval was achieved by steam heating in a solution of citrate buffer, pH 6.0 for 30 minutes. Sections were blocked with 10% normal horse serum for monoclonal antibodies and 10% normal goat serum for polyclonal antibodies. Sections were then incubated overnight with primary antibodies monoclonal mouse anti-human synaptopodin antibody (1∶20) (gift of Dr. Peter Mundel), polyclonal rabbit anti-human podocin antibody (1∶1000) (gift of Dr. Peter Mundel), monoclonal mouse anti-human nephrin antibody (1∶50) (gift of Dr. Karl Tryggvason), and polyclonal rabbit anti-human nephrin antibody (1∶200) (Enzo Life Sciences Inc., Farmingdale, NY) at 4°C. Horse anti-mouse IgG and goat anti-rabbit IgG (Dako Inc. Carpinteria, CA) were used as secondary antibodies (1∶1000) for 30 minutes. The sections were then incubated in avidin-biotin complex at 1∶25 dilution (Vector Labs, Burlingame, CA) and developed using diaminobenzidine (DAB) as chromogen. After washing, the sections were counter-stained with hematoxylin and coverslipped. Negative controls were carried out by incubation in the absence of the primary antibody.

#### Morphometry

Areas of positive DAB staining as a percentage of the entire glomerulus were calculated using the ImageJ software (US National Institutes of Health) for each glomerulus in the biopsy sample. Subsequently, a mean value of positive DAB staining was calculated based on the number of glomeruli for each biopsy sample.

#### Enzyme-linked immunosorbent assay

Urinary nephrin was determined by a competitive enzyme-linked immunosorbent assay, using polyclonal antibodies against the extracellular domain (amino acids 23–322) of human nephrin (Exocell Inc., Philadelphia, PA). The assay was performed by Exocell Inc. according to manufacturer's instructions. Briefly, urine samples were diluted in the range of 1∶10 to 1∶500 (depending on degree of proteinuria in urines sample). A 50-µl diluted sample was added to each well already coated with rat nephrin followed by the addition of a 50-µl of rabbit anti-nephrin antibody for an incubation of 60 minutes at room temperature. Plates were washed followed by the incubation with 100-µl of anti-rabbit HRP conjugate to each well for 60 minutes. After plates were washed and color developed, absorbance was read at 450 nm. Elevated levels of urinary nephrin or nephrinuria was defined as urine nephrin-creatinine ratio (UNCR) (mg/g) ≥0.1 mg/g. This value was based on 10 healthy controls who consistently exhibited UNCR <0.1 mg/g.

### Ethics

Archival human kidney biopsies and random urine samples were collected at Jacobi Medical Center, Bronx, New York as part of exempted protocols “Differential Protein Expression in Nephrotic Diseases” and “Analysis of Urinary Proteins in Nephrotic Syndrome”, both of which were approved by the Institutional Review Board of the Albert Einstein College of Medicine of Yeshiva University. Since all clinical data were de-identified, no consent was required.

### Statistical methods (if applicable)

Analyses were performed comparing study and control biopsies and also between 4 groups defined as normal controls (no diabetes), normo-, micro-, and macroalbuminuria. Categorical variables were presented as percentage while continuous variables were presented as median (interquartile range) or mean ± standard deviation (SD) if normality assumptions were not substantially violated. Differences between mean rank expression of proteins between study and control biopsies were calculated using Mann-Whitney test. Difference in UNCR between groups was determined Kruskal-Wallis. Spearman correlations were calculated to assess trend for continuous variables and Chi-square was used to assess associations of categorical variables. Both UACR and UNCR were log transformed for graphical depiction. Analyses were performed with STATA Version 8.2 and GraphPad Prism Version 5.0 for Windows software and results were considered statistically significant if P<0.05.

## Results

### Synaptopodin, podocin, and nephrin expression are significantly decreased in DN as compared to controls

**Figure 1:**
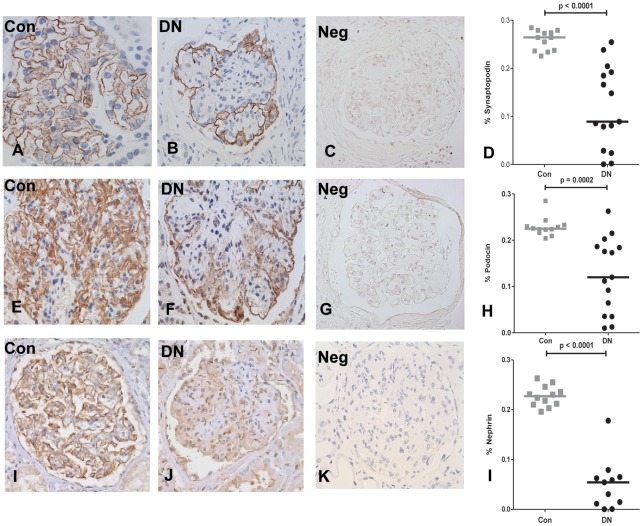
Synaptopodin, podocin, and nephrin are significantly downregulated in diabetic nephropathy (DN) as compared to controls (Con). Representative staining of synaptopodin in Con (A), DN (B), podocin in Con (E) and DN (F), and nephrin in Con (I) and DN (J). Negative controls without administration of primary antibody are represented for synaptopodin (C), podocin (G), and nephrin (K). Quantification of synaptopodin (D), podocin (H), and nephrin (L) positive area per glomerular tuft in Con (12 patients) and DN (15 patients). Horizontal lines represent the median value. *Number of DN biopsies for nephrin was 11 due to lack of remaining tissue.

Clinical characteristics of diabetic and control patients are summarized in Table 1. Of the 15 biopsies, 14 had pathologic findings of moderate to severe nodular diabetic glomerulosclerosis, the remaining included 1 with diffuse diabetic glomerulosclerosis with extensive global glomerulosclerosis. All biopsies showed evidence of both arteriosclerosis and arteriolosclerosis, and 12 biopsies had tubular atrophy and interstitial fibrosis. All but 2 of the diabetic biopsy patients had nephrotic range proteinuria. Control biopsies revealed intense linear staining of synaptopodin, podocin and nephrin in podocytes along the capillary walls (Fig. 1A, E, I). Diabetic biopsies, on the other hand, demonstrated granular, discontinuous staining with areas of absent expression of synaptopodin, podocin, and nephrin (using antibody from Enzo LifeSciences, Inc.) (Fig. 1B, F, J). Consistent results were found when nephrin antibody from Dr. Karl Tryggvason was used (data not shown). When quantified, synaptopodin, podocin, and nephrin expression were significantly decreased in DN, despite exhibiting glomerulomegaly, as compared with control biopsies (Fig. 1D, H, L). Negative controls without administration of primary antibody is represented for each antibody (1, C, G, K).

**Table 1:** Clinical characteristics of control and Type 2 diabetic nephropathy patients.

Clinical Characteristic	Control (n = 12)	Diabetic Nephropathy (n = 15)	P value
Age (years)	56 (52;60)	56 (51.9;63.5)	0.83
Male sex (%)	75%	66%	0.67
Urine protein-to-creatinine ratio (g/g)	–*	5.3 (3.9;7.8)	–
Blood urea nitrogen (mg/dL)	15 (12.8;17.3)	51 (39.5;63.3)	<0.0001
Serum creatinine (mg/dl)	1.0 (0.8;1.1)	4.1 (2.7;4.8)	0.0005
estimated GFR (ml/min/1.73 m^2^)	86.8 (61.8;103.7)	13.35 (12.5;40.7)	0.0008
Serum albumin (g/dl)	4.2 (3.7;4.4)	2.5 (2.3;3.6)	0.0002
Systolic blood pressure mm/Hg	127 (120;130)	150 (140;166)	0.0002
Diastolic blood pressure (mm/Hg)	80.5 (73.4;86.2)	85 (76;91)	0.49
Hb_A1C_%	6.5 (4.5;10.9)	9.3 (7.8;11.8)	0.10
ACEI† (%)	25%	73%	0.026
ARB‡ (%)	8.3%	26%	0.25

Data are presented as median (interquartile range) for continuous variables and % for categorical variables.

* Urine protein-creatinine ratio not available for control patients as their urinalyses did not show proteinuria.

† ACEI: angiotensin converting enzyme inhibitors.

‡ ARB: angiotensin receptor blockers.

### Nephrinuria detected in patients with normoalbuminuria

**Figure 2:**
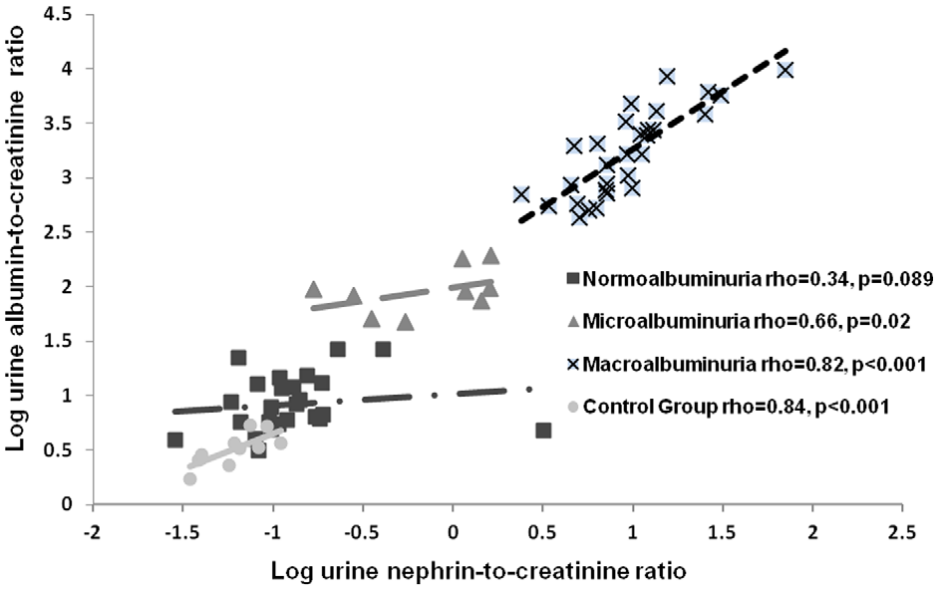
Correlations between urine nephrin-to-creatinine ratio (UNCR) and clinical markers of renal disease. A) Log transformed UNCR correlates significantly with macroalbuminuria (*rho* = 0.82, p<0.0001) and microalbuminuria (*rho* = 0.66, p = 0,02), and not significantly with normoalbuminuria (*rho* = 0.34, p = 0.09) when divided into groups according to level of albuminuria.

We subsequently measured urinary nephrin in 66 diabetic patients of all chronic kidney disease (CKD) stages and levels of albuminuria and compared them to 10 healthy control subjects. Clinical characteristics are summarized in Table 2. There were 26 patients with normoalbuminuria, 11 patients with microalbuminuria, and 29 patients with macroalbuminuria. A significant difference in median urine nephrin-to-creatinine ratio (UNCR) was observed between control subjects and all diabetic categories (p<0.0001) (Table 2). In addition, nephrinuria was detected in 54% of normoalbuminuric patients. UNCR also did not differ significantly from those treated and those not treated with angiotensin converting enzyme inhibitors or angiotensin receptor blockers amongst subgroups of normo-, micro-, and macroalbuminuria (p = 0.68, 0.62, and 0.32 respectively). We found the highest correlation between UNCR levels with albuminuria (*rho* = 0.89, p<0.001), serum creatinine (*rho* = 0.43, p = 0.0002), BUN (*rho* = 0.37, p = 0.001) and systolic blood pressure (*rho* = 0.32, p = 0.007) (Table 3). UNCR showed negative correlations with serum albumin (*rho* = −0.48, p<0.0001) and eGFR (*rho* = −0.33, p = 0.005) (Table 3). UNCR also correlated in subgroups of microalbuminuric (*rho* = 0.66, p = 0.02) and macroalbuminuric (*rho* = 0.82, p<0.001) patients (Figure 2). The correlation in normoalbuminuic patients (*rho* = 0.34, p = 0.09) was not significant despite a trend in that direction.

**Table 2:** Clinical parameters of control and type 2 diabetic patients.

	Control n = 10	Normo-albuminuria n = 26	Micro-albuminuria n = 11	Macro-albuminuria n = 29	P value
Men (%)	40%	44%	45%	53%	0.85
Age	61±12.2	62±14	68±7.3	59±13	<0.0001
HbA1C	–	7.6 (6.5,8.1)	7.2 (6.3,8.0)	7.5 (6.8,9.0)	0.6
UACR* (mg/g)	7.32 (3.7,14.5)	7.9 (5.6,12.8)	93.8 (79.1,185.4)	1644.3 (750.3,3013.3)	<0.0001
Serum creatinine (mg/dL)	0.9 (0.8,1.0)	1.4 (0.9,1.6)	1.8 (1.5,2.1)	2.1 (1.6,2.9)	<0.0001
estimated GFR (ml/min/1.73m^2^)	89 (65,120)	47 (36.2,72.2)	37 (29,48)	30 (20,44.5)	<0.0001
Systolic blood pressure (mmHg)	125 (102,149)	132 (120,145)	139 (129,159)	144 (136,154)	0.0001
Diastolic blood pressure (mmHg)	67 (54,85)	68 (54,76)	70 (64,72)	76 (66,88)	0.21
% Diabetic Retinopathy	–	43%	20%	55%	0.15
UNCR† (mg/g)	0.07 (.03,.098)	0.11(.08,.17)§	1.16 (.35,1.60)	9.15 (5.68,12.08)	<0.0001‡
% ACEI/ARB treatment	0% (0/10)	73% (19/26)	54% (6/11)	62% (18/29)	0.0004‡
% UNCR >0.1 (mg/g)	0% (0/10)	54% (14/26)	100% (11/11)	100% (29/29)	0.001‡

Data are means +/− SD or median (interquartile range).

* UACR: urine albumin-to-creatinine ratio.

† UNCR: urine nephrin-to-creatinine ratio.

‡ P for trend across the 4 categories.

§ P <0.05 vs. Control.

**Table 3:** Correlations of urine nephrin-creatinine ratio (UNCR) with clinical parameters.

Parameter	n value	*Rho*	P value
UACR* (mg/g)	61	0.89	<0.001
Serum creatinine (mg/dL)	65	0.43	0.0002
Blood urea nitrogen (mg/dL)	65	0.37	0.001
estimated GFR (ml/min/1.73m^2^)	65	−0.33	0.005
HbA1C	63	0.20	0.10
Serum albumin (g/dL)	55	−0.48	0.0001
Systolic blood pressure (mmHg)	64	0.32	0.007
Diastolic blood pressure (mmHg)	64	0.21	0.07
Presence of diabetic retinopathy	58	0.14	0.27
Presence of ACEI† or ARB‡	66	−0.04	0.75

Correlations were determined by calculations of Spearman *rho.*

* UACR: urine albumin-to-creatinine ratio.

† ACEI: angiotensin converting enzyme inhibitor.

‡ ARB: angiotensin receptor blocker.

## Discussion

Our results substantiated the dysregulated podocyte phenotype in human DN and quantified expressions of synaptopodin, podocin and nephrin in all Type 2 diabetic biopsies. Decreased expression of protein and mRNA levels has been described in podocyte-associated molecules in both animal models [26]–[29] and human subjects of DN [12], [13], [16]–[18], [30], [31]. Though significant decreased expression of these podocyte-specific proteins may have been anticipated, we found that the extent of loss is not identical, i.e. these three biomarkers are not interchangeable, suggesting that they have different regulatory factors. For instance, synaptopodin is susceptible to cleavage by cathepsin L, a lysosomal enzyme that is induced in DN [19], [32]. Cathepsin L also cleaves the GTPase dynamin, which has been shown to be essential for podocyte function [32]. Whether podocin or nephrin are as vulnerable is unknown at this time.

Furthermore, nephrin may be unique in that it modulates the podocyte's response to insulin. Coward et al. has shown that podocytes that are nephrin deficient results in at state of insulin resistance [33]. Interrupting the fusion of glucose transporters GLUT4 and GLUT1 to the plasma cell membrane when nephrin is absent appears to be the mechanism [33]. There also exists evidence that when podocytes in cell cultures are treated with angiotensin and glycated albumin (insults relevant to diabetes), there is shedding of extracellular domain of the nephrin molecule from podocyte cell surface into the urinary space [17]. Interestingly, insulin resistance in the podocyte alone is adequate to achieve signs of DN, as demonstrated in a knock out mouse model of insulin receptor deficiency under normoglycemia [34]. Therefore, the diabetic milieu may represent a vicious cycle of hyperglycemia, nephrin loss, podocyte insulin resistance, exacerbated hyperglycemia resulting in a severe DN phenotype.

That nephrinuria is seen in early disease (in 54% of patients with normoalbuminuria) and increases in overt disease (macroalbuminuria) supports a previous finding by Patari et al. who had described nephrinuria in one-third of diabetic patients with normoalbuminuria [24]. In contrast to Patari et al., however, is our report of a significant correlation between nephrinuria and albuminuria. Patari et al. has described the presence of similar levels of nephrinuria in all stages of albuminuria, i.e. normo-, micro-, newly discovered micro-, and macroalbuminuria. However, their method of detection for nephrinuria was via Western blotting, which showed either the presence or absence of nephrin fragments. They used an antibody that detected parts of the intra- and extracellular domains of nephrin (amino acids 1031–1055 and 1096–1215). Since the level of nephrinuria was not quantified, it is not immediately obvious whether there would have been an association with albuminuria. We, on the other hand, employed the ELISA method which used an antibody that detected the extracellular domain of nephrin (amino acids 23–322) which allowed for quantification of nephrinuria. Thus it is difficult to compare our results from those of Patari et al. as methodologies of nephrin measurement were different. Nephrinuria has also shown a significant association with lower eGFR among normoalbuminuric patients in a Chinese population, suggesting that these patients are at risk of developing renal insufficiency [35]. Interestingly, a recent study by Zheng et al demonstrated that urinary mRNA of synaptopodin, podocin, and other podocyte-specific molecules increased with progression of DN [36]. Though our study is protein based, our results complement those findings by demonstrating that urinary nephrin also correlates well with disease progression and harbors the potential to be an early biomarker.

### Limitations

There are a few limitations to this study. As renal biopsies are not routinely performed to diagnose DN, these patients may represent a separate category of patients which may skew podocyte expression. For example, they may demonstrate a more severe subset with rapidly progressive renal failure or exhibit concomitant diagnoses with the presence of hematuria. Our biopsies indicate a more advanced group of patients, given that the mean eGFR was 27.2 ml/min/1.73 m^2^. Unfortunately, it is difficult to avoid this limitation as it may be unethical to biopsy all classic presentations of DN, though we used only biopsies that had pathognomonic findings. Also, since the comparison of nephrinuria with clinical data was a cross-sectional study, we do not know if nephrinuria is part of a causal mechanism, or if early nephrinuria will consistently predict subsequent DN. Measuring nephrinuria prospectively in diabetic patients with normoalbuminuria will help to answer these questions, though this type of study will require a prospective study for at least 10–20 years after onset of diabetes.

In conclusion, we have shown significantly reduced expressions of synaptopodin and podocin, and nephrin in DN. As a corollary, we have shown that a high percentage of these patients exhibit nephrinuria without albuminuria, suggesting its potential utility as an early biomarker. If further research confirms nephrinuria to be a biomarker of pre-clinical DN, it would shed light on podocyte metabolism in disease, and raise the possibility of new and earlier therapeutic targets.
